# Is Coaches’ Perceived Team Performance Associated with Training Load and Fatigue in Spanish Semi-Professional Soccer?

**DOI:** 10.3390/jfmk11040375

**Published:** 2026-09-22

**Authors:** Jorge Polo-Tejada, David Lobo-Triviño, Rosa Ayuso-Moreno, Borja Sanabria-Pino, Ana Rubio-Morales, José Carlos Ponce-Bordón

**Affiliations:** 1 Faculty of Sport Sciences, University of Extremadura, 10003 Caceres, Spain; jpolote@unex.es (J.P.-T.); davidlt@unex.es (D.L.-T.); rmayusom@unex.es (R.A.-M.); borjasp@unex.es (B.S.-P.); rubiomorales@unex.es (A.R.-M.); 2 Institute for Research and Innovation in Sport, University of Extremadura, 10003 Caceres, Spain

**Keywords:** football, mental load, monitoring, questionnaire, training

## Abstract

**Objectives**: This study investigated the influence of coaches’ perceived team performance on training load and fatigue across the weekly microcycle in semi-professional soccer players. **Methods**: Twenty-four players were monitored over 183 training sessions during a 34-week competitive period in the Spanish fourth-tier league. External load was assessed using 10 Hz GPS devices, while internal-load-related, mental-load-related variables, and wellness were measured using validated self-report questionnaires. Coaches’ evaluations of team performance following matches were also included as a key variable. **Results**: No statistically significant associations were detected between coach-perceived performance and any external load variable after adjustment for multiple testing. Also, no significant associations were observed between perceived performance and internal or mental load. No significant associations were detected for wellness variables. However, weekly comparisons showed that microcycles following good perceived performance involved lower accumulated external load than those following medium or poor performance. No statistically significant differences were detected between conditions for internal-load-related, mental-load-related variables, or wellness. **Conclusions**: In this single semi-professional male team, lower accumulated external load was observed in microcycles following good perceived performance, whereas no corresponding differences were detected in internal load, mental load, or wellness. These preliminary associations require confirmation in multi-team designs.

## 1. Introduction

Training load (TL) monitoring has become a cornerstone of soccer performance management due to its critical role in optimizing physical performance and reducing injury risk [1,2]. For this purpose, understanding the different types of loads involved in soccer is necessary. External load refers to the physical work performed by players during training sessions and matches [3], while internal load reflects the individual physiological responses elicited by physical exertion [4]. External and internal load monitoring provides a comprehensive understanding of the physical and physiological demands imposed on players throughout the training and competitive season [5]. Beyond physical demands, mental load, defined as the cognitive and emotional demands experienced by athletes during training tasks [6], has emerged as a relevant factor in monitoring load. For instance, training activities performed under greater time pressure tend to elicit higher levels of perceived mental effort compared with tasks that allow for extended decision-making time [7].

How TL influences players’ well-being should also be considered in the monitoring process [8]. Previous research has demonstrated the influence of TL on fatigue, which is considered a complex phenomenon influenced by both physical and psychological factors that may reduce performance and increase the risk of injury [9]. Several methods have been applied to assess fatigue in soccer players [10]. A commonly used tool for fatigue monitoring is a brief wellness questionnaire composed of five items scored on a 5-point Likert scale ranging from 1 to 5 [11]: fatigue, sleep quality, muscle soreness (DOMS), stress, and mood. This questionnaire has been widely applied in soccer [12].

Systematic TL monitoring and fatigue management constitute essential components of performance optimization strategies in soccer. On the other hand, team performance can also be quantified using objective indicators such as match outcomes and final league rankings [13,14]. Although numerous studies have examined these objective indicators of performance, limited research has considered alternative performance measures, such as coaches’ subjective perceptions of team performance, which may be a relevant yet understudied factor [15]. Coaches’ perceived team performance can be defined as the degree of satisfaction with the team’s competitive progression, including absolute performance level, performance improvement, and achievement of season objectives [16]. Previous research has shown that coaches’ evaluative feedback plays a key role in both individual and team performance [17]. However, limited evidence exists regarding the relationship between coaches’ subjective evaluations and teams’ physical performance metrics. For example, Ponce-Bordón et al. [18] examined match running performance and coaches’ performance ratings, reporting only weak associations and indicating that higher coach-rated performance was associated with lower distances covered by professional soccer teams.

Research has reported that TL may be related to several contextual factors, such as the objective outcome of the previous match, with higher training loads reported following a win [19,20]. It is necessary to highlight that match outcome is a different concept from coaches’ perceived team performance. This distinction is important because a coach may assign a low-performance rating to a match that ended in a win if the team did not execute the game plan effectively, or a high rating to a loss against a superior opponent. Therefore, coach-perceived performance quality and objective match outcome represent distinct constructs that may show opposite associations with subsequent training prescription. However, the literature examining TL (i.e., internal, external, and mental load) and fatigue has not deeply explored the interaction between coaches’ perceived team performance and TL across the weekly microcycle. Therefore, this study aimed to examine the relationship between coaches’ perceived team performance and TL and fatigue during the weekly microcycle. Based on previous evidence, the following hypotheses were proposed: H1) higher coach-perceived team performance would be associated with lower subsequent external load, specifically in TD, VHSR, sprint distance, ACC, and DEC [21]; and H2) higher coach-perceived team performance would be associated with more favorable wellness scores, that is, with higher ratings across the six wellness items (fatigue, sleep quality, DOMS, stress, mood, and mental state) [22]. No directional hypothesis was formulated for internal- or mental-load-related variables.

## 2. Materials and Methods

### 2.1. Study Design

This study adopted a prospective, longitudinal, and observational design to examine the association between coaches’ perceived team performance across competitive matches and the subsequent weekly TL. Training-load-related variables, fatigue-related variables, and coach-perceived performance data were systematically collected throughout the 2024/25 competitive season as part of routine team monitoring and subsequently analyzed retrospectively. All training sessions were monitored throughout the season (from 1 September 2024 to 4 May 2025, i.e., just the competitive phase of the season). Only players who completed four or more training sessions per week were included in the analysis in order to reduce between-player variability in weekly exposure [3]. Training sessions were conducted between 10:30 a.m. and 12:30 p.m. under standardized conditions and were systematically monitored to quantify TL and fatigue variables. Player wellness was assessed daily using a self-reported wellness questionnaire completed on arrival at the training facility, before the start of each session. External load was recorded using GPS devices distributed at the start of the training session. Internal and mental load were measured using a validated questionnaire administered individually to players immediately following each training session. Coach-perceived team performance was evaluated using a single-item 1–10 global rating scale completed by the head coach three days after each official match. This time delay was implemented to reduce the potential emotional bias and immediate psychological effects associated with match outcomes, thereby enhancing the objectivity of the subjective evaluation. Figure 1 shows when the different instruments are used to monitor the load.

### 2.2. Sample

The sample was obtained through convenience sampling, comprising all outfield players from a single semi-professional team who met the inclusion criteria (participation in at least four training sessions per week). Thus, a total of 24 semi-professional outfield players (M_age_ = 29.05 ± 4 years old) participated in the study, contributing data from 183 total training sessions monitored, with a total of 2772 observations over a 34-week period during the competitive phase of the 2024/25 season in the Spanish fourth-tier soccer league (Second Federation). Although a larger number of sessions were initially recorded, five training sessions were excluded from the analysis because they corresponded to the first week of the competitive season, during which no prior performance evaluation was available. Likewise, six training sessions conducted during the mid-season league interruption due to the Christmas break were also excluded, as no competitive performance indicators were available for analysis. Goalkeepers were excluded due to the specific tactical and physical demands associated with their playing position, which differ substantially from those of outfield players. Only players who fully completed each monitored training session were included in the analyses. The study protocol was approved by the Ethics Committee of the Universidad de Extremadura (protocol code: 33//2024). All procedures were conducted in accordance with international ethical standards for research involving human participants. Data handling complied with the privacy, confidentiality, and data protection standards by the American Psychological Association.

### 2.3. Instruments and Variables

#### 2.3.1. Coaches’ Perceptions of Team Performance

The coach’s perceived team performance is defined as the coach’s overall assessment of the team’s collective performance during a match, based on the coach’s expert observation of the team’s tactical, technical, and competitive behavior [23]. This type of subjective assessment has been used in sports performance analysis studies as a complementary measure to objective game indicators [24,25]. In the present study, the head coach evaluated the team’s performance three days after each official match using a numerical scale from 1 to 10, where 1 represented very poor performance and 10 represented excellent performance. The score reflected the coach’s overall perception of the team’s collective performance during the match.

For the overall evaluation, the coach considered multiple dimensions of performance: (i) tactical aspects were assessed, including the degree of compliance with the strategic game plan and players’ positioning and rational occupation of space, all of which are considered fundamental to collective performance [26]. (ii) Technical aspects were evaluated, such as passing accuracy, ball control, shooting effectiveness, and crossing quality. Efficient technical execution is directly associated with improved competitive performance and a reduction in critical in-game errors [27]. (iii) Psychological and attitudinal variables were also included in the assessment, such as concentration level, tactical discipline, resilience following adverse situations, and overall competitive attitude. Scientific evidence indicates that these factors modulate sports performance and performance stability under pressure [28]. (iv) Decision-making in real game situations was evaluated, as well as the ability to adapt decisions to the competitive context of the match. Decision-making quality is a key component of expert performance in team sports [29].

Then, to measure the influence of a coach’s perceived team performance on subsequent TL and fatigue, team performance was categorized into three groups based on terciles: bad performance (i.e., training microcycles after coach’s scores of 1–6; *n* = 1213 observations from 78 training sessions conducted), medium performance (i.e., training microcycles after coach’s score of 7; *n* = 853 observations from 50 training sessions conducted), and good performance (i.e., training microcycles after coach’s scores of 8–10; *n* = 706 observations from 44 training sessions conducted). This variable was calculated without considering the training sessions from the first week (as there were no previous official matches) and the winter break (as there were no official matches). Finally, it is necessary to highlight that coach-perceived performance modeled as a continuous score of 1–10 constituted the primary exposure of interest, whereas the tercile-based categorization was used for secondary, exploratory between-microcycle comparisons. For these secondary models, the effective unit of analysis was the microcycle (*n* = 33), since all players within a given microcycle shared the same coach performance rating.

#### 2.3.2. External Load

External load variables were recorded using the Polar Team Pro GPS system (Polar Electro Oy, Kempele, Finland; version 1.4.2). This technology has been validated for use in team sports, allowing the monitoring and quantification of physical demands through specific sensors [30,31]. Each unit is equipped with a triaxial accelerometer and operates at a sampling frequency of 10 Hz. A heart rate monitor is also included. Each player wore their personal GPS unit using an adjustable chest strap positioned at the sternum level, approximately five minutes before the start of each training session. Specifically, the variables recorded were total distance covered by soccer players in meters (TD), distance covered between 21 and 24 km·h^−1^ (very high-speed running; VHSR), distance covered above 24 km·h^−1^ (sprint), number of accelerations performed above 3 m·s^−2^ (ACC), and number of decelerations performed below −3 m·s^−2^ (DEC).

#### 2.3.3. Internal Load

Internal load variables were recorded through quantification of the rating of perceived exertion (RPE) using the 10-point Borg scale [32]. At the end of each training session, players were asked the question: “How hard did you feel you worked during the session?” They responded by assigning a numerical value between 0 and 10, where 0 represented the lowest level of exertion and 10 represented the highest level of exertion. This method has been previously validated as a reliable measure for monitoring internal TL in team sports [33]. Session-RPE (sRPE, au) was also subsequently computed as the product of the RPE value and the duration of the session in minutes and was used as an indicator of accumulated internal load [32,33].

#### 2.3.4. Mental Load

Mental-load-related variables were assessed using an adapted version of the Questionnaire for the Assessment of Mental Load in Team Sports (CCMDE), previously validated in team sport contexts [6]. Following the completion of each training session, players were required to rate their perceived cognitive demand, emotional demand, and individual satisfaction. Additionally, subjective mental fatigue was measured using a 0–10 visual analog scale (VAS), widely applied in soccer research for the assessment of perceptual fatigue responses [7]. Each dimension was evaluated using a 10-point Likert scale ranging from 0 (lowest perceived demand) to 10 (highest perceived demand).

#### 2.3.5. Wellness Variables

Finally, player wellness variables were collected using a self-reported questionnaire completed before the start of each training session. The questionnaire consisted of five items assessing fatigue, sleep quality, muscle soreness, stress, and mood. Each item was rated using a five-point Likert scale with one-point intervals, ranging from 1 to 5, where 1 indicated a very poor state and 5 indicated a very good state [11]. This questionnaire was modified by adding an additional item assessing mental fatigue, defined as a psychobiological state characterized by feelings of tiredness and a lack of energy, induced by prolonged periods of demanding cognitive activity [34]. An additional item assessing mental state was included in the questionnaire (‘What is your level of mental fatigue related to the previous training session?’). Mental state was defined as a state of tiredness resulting from increased cognitive demands [35]. This item was also rated using the same 5-point Likert scale, ranging from 1 to 5. To ensure consistency with the overall wellness score, responses were coded so that higher scores reflected better perceived mental well-being and lower levels of mental fatigue.

### 2.4. Statistical Analysis

Data analysis was performed using RStudio (version 2024.12.0+467) and the dplyr, tidyr, and psych packages for data manipulation and statistical calculations.

First, linear mixed models (LMMs) were applied to examine the influence of coaches’ perceived team performance on TL and fatigue variables. LMMs have demonstrated their ability to function with unbalanced and repeated-measures data [36]. For instance, TL and fatigue variables in training sessions are stored for players (i.e., each player has a record for every training session they have participated in, and each training session has observations of several players). Thus, a two-level hierarchy was modeled for the analysis. External load (i.e., TD, VHSR, sprint, ACC, and DEC); internal and mental load-related variables (i.e., RPE, cognitive, emotional, mental fatigue, and satisfaction); and fatigue (i.e., fatigue, sleep, DOMS, stress, mood and mental state) variables were included as dependent variables in the models, and coach’s perceived team performance was included as a predictor variable of TL and fatigue. Player and match-week were included as random intercepts in the analysis. Models were estimated using the lmer function from the lme4 package. To account for multiple testing across the 17 outcome variables, *p*-values for the coach’s perceived team performance coefficients were adjusted using the Benjamini–Hochberg false discovery rate (FDR) procedure. Model assumptions (linearity, homoscedasticity, and normality of residuals) were visually inspected using standard diagnostic procedures.

Gaussian linear mixed models were retained for all outcomes. Acceleration and deceleration counts had to be sufficiently large for each session for the normal approximation to be reasonable, and five-category ordinal wellness items were robustly analyzed with parametric models for the present sample sizes [37]. Full residual diagnostics are reported in Supplementary Table S1; residual distributions for several wellness items were negatively skewed and leptokurtic, and the corresponding estimates should therefore be interpreted with additional caution.

Second, LMMs were again performed to measure the differences between weeks according to the coach’s perceived team performance and subsequent weekly TL and fatigue. The total weekly external load was calculated by the sum of daily averages to obtain a weekly total load for each variable. The weekly internal and mental load and fatigue were also calculated by averaging daily averages to obtain a weekly average load for these variables. This approach provided an overall view of the workload over the course of the week. Then, the sum of external load (i.e., TD, VHSR, sprint, ACC, and DEC), the average internal and mental load (i.e., RPE, cognitive, emotional, mental fatigue, and satisfaction), and fatigue (i.e., fatigue, sleep, DOMS, stress, mood and mental state) variables were included as dependent variables in the models, and different types of weeks (i.e., bad performance, medium performance, and good performance) were independent variables included as fixed effects. The variables player and match-week were also considered as random effects in the analysis. Values were represented as coefficients and standard errors (coeff ± SE). Additionally, post hoc comparisons were performed using the emmeans package, with Tukey adjustment for multiple comparisons, to obtain marginal means of the dependent variables for each type of week and to compare differences between them. Statistical significance was set at *p* < 0.05. Finally, Cohen’s effect sizes (ES) [38] were also calculated to quantify the magnitude of differences observed in between-week comparisons. The thresholds for effect size interpretation were set as follows: trivial (<0.20), small (0.20–0.59), moderate (0.60–1.19), large (1.20 1.99), very large (2.00–3.99), and extremely large (>4.00).

Finally, a simulation-based sensitivity analysis was performed using the simr package to determine the magnitude of the team performance effect that could be detected with 80% power under the observed multilevel design (Supplementary Table S2). Also, model diagnostics were calculated to examine the convergence, residual assumptions, missing-data procedures, and random-effect variance of models included in the analysis (Supplementary Table S1).

## 3. Results

The association between coach-perceived team performance (continuous score; primary analysis) and external load variables is presented in Table 1. No statistically significant association was detected for any variable. The largest point estimate corresponded to sprint distance (β = −0.86, SE = 1.11, 95% CI [−3.04, 1.32], *p* = 0.444), with a confidence interval including zero.

Table 2 shows the influence of the coach’s perceived team performance on internal and mental load-related variables. The model showed that performance was not significantly associated with any variables.

The results for wellness variables are presented in Table 3. No statistically significant association was detected for any variable. The point estimates for sleep quality (β = 0.02, SE = 0.01, 95% CI [−0.007, 0.042], *p* = 0.175) and stress (β = 0.02, SE = 0.01, 95% CI [−0.008, 0.039], *p* = 0.195) were positive but small, with confidence intervals including zero.

Differences in weekly accumulated external load during the following sessions of the microcycle according to perceived team performance are presented in Table 4. Overall, after weeks with medium team performance, soccer players exhibited the highest values across most variables (i.e., TD, VHSR, sprint, ACC, and DEC), followed by bad team performance, whereas good performance weeks consistently showed the lowest values. Specifically, differences were observed between medium and good team performance in TD (*p* < 0.05; ES = 0.43), VHSR (*p* < 0.01; ES = 0.55), sprint (*p* < 0.001; ES = 0.84), ACC (*p* < 0.001; ES = 0.87), and DEC (*p* < 0.01; ES = 0.57), indicating lower external weekly load after good team performance. Additionally, differences between bad and good team performance were found for sprint (*p* < 0.001; ES = 0.71), ACC (*p* < 0.01; ES = 0.44), and DEC (*p* < 0.05; ES = 0.33), with higher values after bad team performance. Differences between bad and medium team performance were also identified for ACC (*p* = 0.01; ES = −0.43), with lower values following bad compared to medium perceived performance.

Table 5 shows the comparisons of internal-load-related and mental-load-related variables during the following sessions of the microcycle across team performance levels. No statistically significant differences were observed between loads for bad, medium, and good performance weeks for RPE, cognitive load, emotional load, mental fatigue, or individual satisfaction. Effect sizes for all between-microcycle comparisons were trivial (|ES| ≤ 0.16), with confidence intervals including zero in every case.

Finally, differences in wellness variables during the following sessions of the microcycle according to team performance levels are presented in Table 6. No significant differences were found between bad, medium, and good performance levels for fatigue, sleep quality, DOMS, stress, mood, or mental state. Effect sizes for all between-microcycle comparisons were trivial to small (|ES| ≤ 0.29), with confidence intervals including zero in every case.

## 4. Discussion

The primary aim of this study was to investigate the influence of coaches’ subjective perceptions of team performance on the subsequent TL (i.e., external-, internal-, and mental-load-related variables) and fatigue during the competitive season in a semi-professional soccer team. The findings of the present study indicated that coaches’ subjective perceptions of team performance were associated with variations in external load during the subsequent weekly microcycle. However, in the primary continuous analysis, these associations did not remain statistically significant after Benjamini–Hochberg correction. The between-microcycle differences reported in the secondary categorical analysis were obtained using Tukey-adjusted post hoc comparisons and should be regarded as exploratory. Therefore, these findings should be considered preliminary and interpreted cautiously. Nevertheless, they provide initial evidence warranting further investigation into whether coaches’ subjective evaluations of team performance may contribute to subsequent training-load prescription and monitoring.

Our results provide only partial and tentative support for the first hypothesis (H1). In the primary continuous analysis, no statistically significant association was detected with any external load variable, although the point estimate for sprint distance was negative and consistent with the hypothesized direction. In the secondary categorical analysis, lower accumulated external load was observed in microcycles following good perceived performance. The internal and mental load models likewise yielded no statistically significant associations, with confidence intervals wide enough to remain compatible with small associations in either direction. For wellness, the point estimates for sleep quality and stress were positive but small and imprecise and did not reach statistical significance.

Regarding the specific differences in coaches’ perceived team performance in categorized groups, matches characterized by good perceived performance were followed by lower values for TD, VHSR, sprint, ACC, and DEC during the subsequent microcycle compared with medium perceived team performance. Additionally, sprint, ACC, and DEC also showed lower values after coaches’ perceptions of good team performance compared with perceptions of bad performance. The observed findings provide a more nuanced understanding of how coaches’ perceptions of team performance may be related to training prescription. Specifically, the fact that these external load variables were lower during the weeks after ‘good’ perceived performance suggests a load management pattern in which high-intensity actions (e.g., sprints, accelerations, and decelerations) were lower following good perceived performance and higher following poor perceived performance [39,40].

On the one hand, the observed reduction in external load following matches perceived positively is consistent with a possible recovery-oriented training response, prioritizing recovery and morale [41]. On the other hand, the higher mechanical demands (i.e., sprints, accelerations, and decelerations) observed after ‘bad’ or ‘medium’ performances are consistent with coaches prescribing more demanding sessions when dissatisfied with competitive outcomes, although the mechanism underlying this association was not directly assessed. This pattern is consistent with the possibility that coaches prescribe higher TL as a corrective response to poor competitive outcomes [21]. While the observed associations between coach-perceived performance and external load are consistent with a reactive training prescription response, several alternative explanations should also be considered. Several context-related variables such as match outcome, player participation in the preceding match, microcycle day, session duration, fixture congestion, opponent quality or stage of the season may independently influence subsequent external training load and were not controlled for in the present models. For instance, matches perceived as poor performances may coincide with periods of particularly demanding fixture schedules or weaker opponents, which could independently contribute to lower subsequent training loads irrespective of the coach’s subjective evaluation. Similarly, seasonal periodization, whereby training load typically follows a planned progressive overload structure, may confound the observed associations.

Interestingly, while external load seemed to be associated with coaches’ perceived performance, RPE and mental load showed no significant associations. This suggests that while physical work was higher in microcycles after matches with dissatisfied coach evaluations, players’ perceived exertion did not necessarily follow the same linear pattern, indicating a potential disconnect between the prescribed physical volume and the players’ subjective experience of intensity, which may also be affected by session duration [42,43]. External load and RPE are moderately to very strongly correlated in soccer, particularly when volume-based GPS metrics are used [44,45,46]. However, the lack of alignment found in our study is consistent with previous research showing that RPE often leaves large unexplained variances for a given external dose and is driven more by total volume than by specific intensity metrics [45,47]. For instance, Mancini et al. [48] suggested that physical performance metrics and cognitive-perceptual performance indicators are not necessarily aligned, supporting the notion that objective physical load alone may not fully capture the complexity of players’ subjective experience of training intensity. Furthermore, research suggests that RPE and coach-player expectations often diverge most during extreme exertion values, where psychological and contextual factors, such as the coach’s satisfaction with performance, shape the player’s perception [49].

Regarding the second hypothesis (H2), the results provided partial support. The LMMs yielded small positive point estimates for sleep quality and stress (β = 0.02), but neither reached statistical significance and both confidence intervals included zero. No significant differences were observed between ‘bad’, ‘medium’, and ‘good’ periods for any wellness variable. These results indicate that no association was detected rather than that player well-being was unaffected; given the precision of the estimates, small associations cannot be ruled out. This stability is consistent with previous research indicating that subjective wellness markers often require a substantial and prolonged accumulation of load to show meaningful clinical changes [50].

Finally, the discrepancy between the continuous and categorical analyses warrants comment. The linear model treats the association between coach-perceived performance and training load as a uniform, dose–response relationship across the full rating scale. In contrast, the categorical comparisons reveal that the most pronounced differences emerge when contrasting the good performance category against the medium and bad categories, suggesting a possible threshold effect at the upper end of the rating scale rather than a consistent linear association. The asymmetric category boundaries (with medium performance defined as a single score of seven) may further amplify between-group differences in the categorical analysis. Readers should interpret the categorical findings as exploratory and consider that the choice of categorization boundaries may influence the observed results.

### Limitations and Future Directions

Despite the longitudinal nature of this study, several limitations should be acknowledged. First, while this study focused on the head coach’s perspectives, it did not account for the perceptions of the entire coaching staff (e.g., assistant coaches, strength and conditioning coaches or analysts) or the players themselves. Although individual satisfaction data were collected, a direct comparison between coaches’ evaluations and players’ self-perceived performance was not the primary focus of this analysis. Future studies should explore the “perceptual gap” between staff and players, as a mismatch in performance assessment could lead to friction in TL acceptance and overall team dynamics.

Second, tactical and technical variables could be included to examine how team performance influences other aspects of the game, including whether poor performance leads to an increase in errors, thereby making it more difficult to win subsequent matches and potentially contributing to a negative streak within the team.

A further limitation was the absence of adjustment for the contextual variables discussed above, which were not available in the present monitoring dataset. Future research should incorporate them as covariates to isolate the association between coach-perceived performance and training load from other contextual influences. Finally, the sample was limited to 24 semi-professional outfield players from a single team, and coaches’ perceived team performance was assessed by a single head coach. Excluding goalkeepers and focusing on a specific competitive level (Spanish fourth tier) may limit the generalizability of these findings. Also, no formal a priori power analysis was conducted. Therefore, the study was based on all available monitoring data from the competitive season, and the findings should be interpreted with caution given the sample size and single-team design. Future investigations should replicate these analyses across multiple teams, coaches, competitive levels, cultural settings and include larger cohorts across different professional levels, gender categories, and academies to establish the external validity of these findings. In addition, primary exposure was measured with a single-item 1–10 global rating scale that has not undergone formal psychometric validation. Although practitioner-derived global ratings have precedent as complementary measures in performance analysis research [24,25] and the construct definition follows previously published frameworks [23], the absence of established reliability and validity evidence for this specific scale limits the interpretation of the findings.

## 5. Conclusions

The results of this study are specific to a single semi-professional male team with a single head coach competing within the Spanish fourth-tier league during the 2024/25 season. Caution is therefore advised in generalizing these results to other teams, competitive levels, gender categories, or coaching contexts. Overall, the main findings suggest that coaches’ subjective perceptions of team performance may be related to subsequent external load in a semi-professional soccer team. Specifically, matches perceived as involving good team performance tended to be followed by lower physical demands during the subsequent microcycle. However, in the primary continuous analysis, these associations did not remain statistically significant after adjustment for multiple testing and should therefore be interpreted cautiously. Although this pattern may indicate that technical staff adjust subsequent training demands according to their perception of previous match performance, further research is needed to determine whether such adjustments represent a deliberate or intuitive corrective strategy.

A dissociation was also observed between the variation in external load and the players’ internal and mental responses. While physical demands fluctuated based on the coach’s perceived performance, no statistically significant associations were detected for players’ internal load, mental fatigue, or overall wellness across performance levels. These non-significant findings should not be interpreted as definitive evidence of an absence of effect but rather suggest that any associations were not detectable at the level of statistical power available.

Given that coaches’ subjective evaluations may inform training prescription, they could be complemented with objective monitoring tools to ensure that physical loads are optimized for performance rather than driven by emotional or tactical dissatisfaction. Future research should continue to explore the perceptual gap between coaches and players to foster a more integrated approach to load management and performance optimization. In addition, future research should objectively measure performance using tactical and technical variables (i.e., shots on goal, move to receive, etc.) to identify discrepancies between subjective perception and objective data.

### Practical Applications

The following considerations derive from a single semi-professional male team evaluated by one head coach during a single season and should be regarded as context-specific rather than as general recommendations for semi-professional soccer.

The findings of this study provide several practical implications for coaches and practitioners in semi-professional soccer. Primarily, there is a clear need to decouple the coach’s perceived team performance from the objective planning of the training microcycle. Since lower perceived performance was associated with an increase in external load metrics, practitioners should be cautious of “corrective” loading. Sudden spikes in these variables have been associated with increased injury risk in professional soccer [51], although neither workload ratios nor injury incidence were assessed here.

To mitigate this, coaching staff should implement a shared decision-making process where subjective variables are cross-referenced with objective GPS data and player wellness profiles. Relying solely on the head coach’s perception may lead to an over-prescription of physical work that does not align with the players’ actual physiological readiness, as suggested by the dissociation between external load and the internal and mental load markers, for which no significant differences were detected. Although this effect was not observed or measured in this study, future research should directly evaluate these hypotheses using longitudinal fitness testing data.

Furthermore, a more balanced approach is necessary to avoid detraining during periods of high perceived performance. The results showed that “good” perceived performance weeks were associated with lower volumes of high-intensity actions. If a coach consistently prescribes lower loads when satisfied with results, the team might fail to maintain the necessary physical stimuli required for long-term competition, potentially leading to a drop in physical capacity later in the season. Therefore, maintaining consistent and objective training stimulus, regardless of match outcomes or tactical satisfaction, is essential for optimizing both health and performance [52].

## Figures and Tables

**Figure 1:**
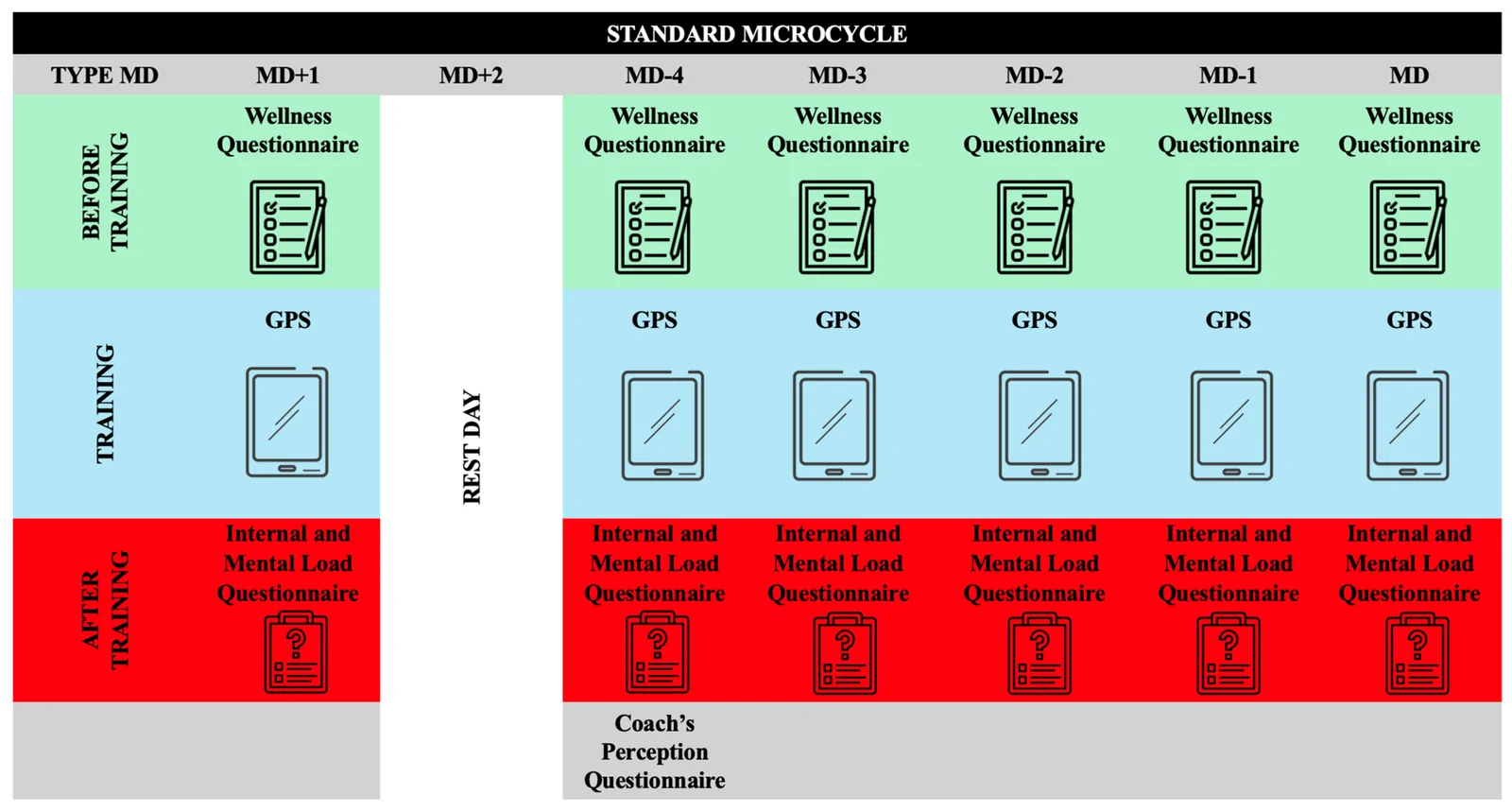
An in-season weekly program for a male soccer team during a standard microcycle.

**Table 1:** Relationship between coach’s perceived team performance and external training load.

	TD (m)		VHSR (m)		Sprint (m)		ACC (No.)		DEC (No.)	
	Coeff	SE	Coeff	SE	Coeff	SE	Coeff	SE	Coeff	SE
Fixed Effects										
Intercept	3690.59 ***	120.88	52.69 ***	2.93	25.76 ***	2.12	12.69 ***	0.71	10.88 ***	0.62
Slope (Team Performance)	23.94	66.65	0.51	1.26	−0.86	1.11	−0.15	0.28	−0.08	0.24
95% CI	(−106.689, 154.565)		(−1.967, 2.989)		(−3.040, 1.317)		(−0.704, 0.396)		(−0.543, 0.392)	
*p*	0.722		0.689		0.444		0.587		0.754	
FDR-adjusted *p*-value	0.889		0.889		0.889		0.889		0.889	

Note. Coeff = coefficient; SE = standard error; m = meters; TD = total distance; VHSR = very high-speed running distance; ACC = number of accelerations; DEC = number of decelerations; *p*-values for team performance coefficients were adjusted for multiple comparisons using the Benjamini–Hochberg false discovery rate (FDR) procedure; *** *p* < 0.001.

**Table 2:** Relationship between coach’s perceived team performance and internal and mental load-related variables.

	RPE		sRPE (au)		Cognitive		Emotional		Mental Fatigue		Individual Satisfaction	
	Coeff	SE	Coeff	SE	Coeff	SE	Coeff	SE	Coeff	SE	Coeff	SE
Fixed Effects												
Intercept	4.67 ***	0.15	354.39 ***	13.88	3.68 ***	0.28	3.24 ***	0.28	2.90 ***	0.35	6.03 ***	0.28
Slope (Team Performance)	0.01	0.05	−1.72	6.22	−0.03	0.05	−0.00	0.03	−0.04	0.04	−0.03	0.02
95% CI	(−0.056, 0.105)		(−13.920, 10.470)		(−0.127, 0.066)		(−0.069, 0.060)		(−0.119, 0.041)		(−0.075, 0.012)	
*p*	0.881		0.784		0.542		0.901		0.341		0.171	
FDR-adjusted *p*-value	0.901		0.889		0.889		0.901		0.889		0.889	

Note. Coeff = coefficient; SE = standard error; RPE = rate of perceived exertion; sRPE = session rating of perceived exertion; au = arbitrary units; *p*-values for team performance coefficients were adjusted for multiple comparisons using the Benjamini–Hochberg false discovery rate (FDR) procedure; *** *p* < 0.001.

**Table 3:** Relationship between coach’s perceived team performance and wellness variables.

	Fatigue		Sleep		DOMS		Stress		Mood		Mental State	
	Coeff	SE	Coeff	SE	Coeff	SE	Coeff	SE	Coeff	SE	Coeff	SE
Fixed Effects												
Intercept	3.85 ***	0.07	4.02 ***	0.10	3.86 ***	0.08	4.24 ***	0.08	4.36 ***	0.09	4.21 ***	0.08
Slope (Team performance)	0.01	0.02	0.02	0.01	0.02	0.02	0.02	0.01	0.01	0.01	0.01	0.01
95% CI	(−0.023, 0.051)		(−0.007, 0.042)		(−0.001, 0.046)		(−0.008, 0.039)		(−0.014, 0.028)		(−0.010, 0.030)	
*p*	0.478		0.175		0.289		0.195		0.515		0.343	
FDR-adjusted *p*-value	0.889		0.889		0.889		0.889		0.889		0.889	

Note. Coeff = coefficient; SE = standard error; DOMS = delayed-onset muscle soreness; *p*-values for team performance coefficients were adjusted for multiple comparisons using the Benjamini–Hochberg false discovery rate (FDR) procedure; *** *p* < 0.001.

**Table 4:** Differences in external load variables according to coach’s perceived team performance level.

	Bad Performance	Medium Performance	Good Performance	*p*	Between-Weeks Comparison (ES)		
	Coeff (SE)	Coeff (SE)	Coeff (SE)		Bad vs. Medium	Bad vs. Good	Medium vs. Good
TD (m)	16,162 (353)	17,006 (447)	15,652 (423)	c *	−0.27 (−0.57, 0.02)	0.17 (−0.12, 0.45)	0.43 (0.10, 0.77)
VHSR (m)	227 (11.2)	253 (13.4)	208 (12.8)	c **	−0.32 (−0.61, −0.02)	0.24 (−0.05, 0.52)	0.55 (0.21, 0.89)
Sprint (m)	121.6 (6.83)	129.9 (8.76)	77.9 (8.27)	b ***, c ***	−0.13 (−0.43, 0.16)	0.71 (0.42, 1.00)	0.84 (0.50, 1.17)
ACC (nº)	54.1 (2.54)	60.4 (2.86)	47.7 (2.77)	a *, b **, c ***	−0.43 (−0.73, −0.13)	0.44 (0.15, 0.73)	0.87 (0.53, 1.22)
DEC (nº)	48.4 (2.46)	51.8 (2.77)	43.7 (2.68)	b *, c **	−0.24 (−0.54, 0.06)	0.33 (0.04, 0.62)	0.57 (0.23, 0.91)

Note. Coeff = coefficient; SE = standard error; ES = effect size; TD = total distance; m = meters; VHSR = very high-speed running distance; ACC = number of accelerations; DEC = number of decelerations; a = significant differences between bad and medium performance; b = significant differences between bad and good performance; c = significant differences between medium and good performance; * *p* < 0.05; ** *p* < 0.01; *** *p* < 0.001.

**Table 5:** Differences in internal-load-related and mental-load-related variables according to team performance level.

	Bad Performance	Medium Performance	Good Performance	*p*	Between-Weeks Comparison (ES)		
	Coeff (SE)	Coeff (SE)	Coeff (SE)		Bad vs. Medium	Bad vs. Good	Medium vs. Good
RPE	4.71 (0.17)	4.76 (0.19)	4.84 (0.18)	ns	−0.05 (−0.36, 0.27)	−0.13 (−0.43, 0.17)	−0.08 (−0.43, 0.27)
sRPE (au)	363.20 (20.25)	356.30 (24.84)	360.85 (24.65)	ns	0.09 (−0.62, 0.80)	0.03 (−0.68, 0.74)	−0.06 (−0.86, 0.74)
Cognitive	3.72 (0.27)	3.68 (0.29)	3.71 (0.29)	ns	0.05 (−0.27, 0.36)	0.01 (−0.29, 0.32)	−0.03 (−0.39, 0.33)
Emotional	3.23 (0.29)	3.33 (0.30)	3.28 (0.29)	ns	−0.12 (−0.44, 0.20)	−0.06 (−0.36, 0.25)	0.06 (−0.29, 0.42)
Mental Fatigue	2.99 (0.34)	2.96 (0.36)	2.94 (0.35)	ns	0.03 (−0.29, 0.35)	0.04 (−0.26, 0.35)	0.01 (−0.34, 0.37)
Individual Satisfaction	6.07 (0.32)	6.00 (0.33)	5.93 (0.33)	ns	0.08 (−0.24, 0.40)	0.16 (−0.15, 0.46)	0.08 (−0.28, 0.44)

Note. Coeff = coefficient; SE = standard error; ES = effect size; RPE = rate of perceived exertion; ns = non-significant differences.

**Table 6:** Differences in wellness variables according to team performance level.

	Bad Performance	Medium Performance	Good Performance	*p*	Between-Weeks Comparison (ES)		
	Coeff (SE)	Coeff (SE)	Coeff (SE)		Bad vs. Medium	Bad vs. Good	Medium vs. Good
Fatigue	3.78 (0.08)	3.78 (0.09)	3.88 (0.09)	ns	0.01 (−0.31, 0.33)	−0.28 (−0.58, 0.03)	−0.29 (−0.64, 0.07)
Sleep	3.96 (0.11)	3.97 (0.11)	4.04 (0.11)	ns	−0.02 (−0.34, 0.30)	−0.23 (−0.54, 0.07)	−0.21 (−0.57, 0.15)
DOMS	3.81 (0.09)	3.80 (0.09)	3.87 (0.09)	ns	0.00 (−0.32, 0.32)	−0.19 (−0.50, 0.12)	−0.19 (−0.55, 0.16)
Stress	4.20 (0.09)	4.20 (0.09)	4.22 (0.09)	ns	−0.00 (−0.33, 0.31)	−0.08 (−0.40, 0.22)	−0.08 (−0.44, 0.28)
Mood	4.34 (0.10)	4.34 (0.10)	4.34 (0.10)	ns	0.02 (−0.30, 0.34)	0.02 (−0.29, 0.33)	0.00 (−0.36, 0.36)
Mental State	4.20 (0.09)	4.18 (0.09)	4.22 (0.09)	ns	0.07 (−0.24, 0.40)	−0.05 (−0.37, 0.25)	−0.13 (−0.49, 0.22)

Note. Coeff = coefficient; SE = standard error; ES = effect size; DOMS = delayed-onset muscle soreness; ns = non-significant differences.

## Data Availability

The data presented in this study are available on request from the corresponding author.

## Supplementary Materials

The following supporting information can be downloaded at https://www.mdpi.com/article/10.3390/jfmk11040375/s1. Table S1. Model diagnostics for dependent variables; Table S2. Simulation-based sensitivity analysis: statistical power to detect the observed team performance effect for each outcome variable.
