# Supplementary material for: Is Coaches’ Perceived Team Performance Associated with Training Load and Fatigue in Spanish Semi-Professional Soccer?
Source: J Funct Morphol Kinesiol. 2026 Sep 22;11(4):375. doi: 10.3390/jfmk11040375 (PMC13692409; doi:10.3390/jfmk11040375)
Supplement: Supplementary file 1 [file jfmk-11-00375-s001.zip › jfmk-4428568-supplementary.pdf]

Supplementary material

Table S1. Model diagnostics for dependent variables.

Outcome	N	Players	Week variance	Residual variance	ICC	Max gradient	Convergence	Residual skewness	Excess kurtosis	Missing (%)
TD	2025	23	375,625.821	2,762,300.35	0.017	0.00	Yes	0.234	-0.698	6.467
VHSR	2025	23	111.321	2351.824	0.036	0.00	Yes	0.057	0.523	6.467
Sprint	2025	23	97.715	1153.385	0.017	0.00	Yes	1.594	1.716	6.467
ACC	2025	23	6.517	56.572	0.108	0.00	Yes	0.396	-0.254	6.467
DEC	2025	23	4.481	54.511	0.096	0.00	Yes	0.497	-0.377	6.467
RPE	1607	23	0.138	4.170	0.071	0.00	Yes	0.234	-0.855	25.774
Session*RPE	1607	23	2819.757	37,760.441	0.038	0.00	Yes	0.484	-0.587	25.774
Cognitive	1607	23	0.179	2.176	0.418	0.00	Yes	0.596	0.832	25.774
Emotional	1607	23	0.060	1.896	0.467	0.00	Yes	1.193	2.662	25.774
Mental Fatigue	1607	23	0.106	2.248	0.537	0.00	Yes	0.870	2.529	25.774
Individual Satisfaction	1607	23	0.004	1.908	0.474	0.00	Yes	-1.092	4.255	25.774
Fatigue	1702	22	0.023	0.527	0.152	0.00	Yes	-1.055	1.698	21.386
Sleep	1702	22	0.007	0.364	0.354	0.00	Yes	-1.196	2.394	21.386
DOMS	1702	22	0.012	0.449	0.224	0.00	Yes	-0.919	1.302	21.386
Stress	1702	22	0.009	0.210	0.412	0.00	Yes	-1.503	5.880	21.386
Mood	1702	22	0.005	0.241	0.427	0.00	Yes	-2.024	8.639	21.386
Mental State	1702	22	0.005	0.235	0.373	0.00	Yes	-1.161	5.592	21.386

Note. All models were evaluated for convergence, singularity, residual distribution, and homoscedasticity. Residual and random-effect distributions were additionally inspected using Q-Q plots, and residuals versus fitted values were examined for systematic patterns. Missing observations were handled using model-specific complete-case analysis without imputation. Week variance represents the variance of the week-level random intercept, and ICC represents the intraclass correlation coefficient.

Table S2. Simulation-based sensitivity analysis: statistical power to detect the observed team performance effect for each outcome variable.

Variable	Power (%)	Lower 95% CI	Upper 95% CI
Fatigue	98.1	97.05	98.85
VHSR	96.0	94.59	97.13
Sleep	96.0	94.59	97.13
Mood	88.9	86.79	90.78
DOMS	87.5	85.29	89.49
Sprint	80.2	77.59	82.63
Mental State	76.4	73.64	79.00
DEC	37.3	34.29	40.38
TD	33.5	30.58	36.52
ACC	28.6	25.82	31.51
Mental Fatigue	25.1	22.44	27.91
Stress	23.8	21.19	26.56
Cognitive	22.8	20.23	25.53
Emotional	9.2	7.48	11.16
Individual Satisfaction	7.6	6.03	9.42
RPE	7.4	5.85	9.20
Session*RPE	32.1	30.50	38.45

Note. Power estimates were obtained from 1000 Monte Carlo simulations using the simr package, based on the observed effect size and the multilevel structure of the present dataset. CI = confidence interval.
